# Murine Splenic Natural Killer Cells Do Not Develop Immunological Memory after Re-Encounter with *Mycobacterium bovis* BCG

**DOI:** 10.1371/journal.pone.0152051

**Published:** 2016-03-21

**Authors:** Mamoru Kawahara, Nozomi Hasegawa, Hiroshi Takaku

**Affiliations:** 1 Research and Development Department, Japan BCG Laboratory, Matsuyama, Kiyose, Tokyo, Japan; 2 Department of Life and Environmental Sciences, Chiba Institute of Technology, Tsudanuma, Narashino, Chiba, Japan; 3 Research Institute, Chiba Institute of Technology, Tsudanuma, Narashino, Chiba, Japan; Public Health England, UNITED KINGDOM

## Abstract

Several lines of evidence have recently suggested that natural killer (NK) cells develop immunological memory against viral infections. However, there is no apparent evidence that NK cells acquire specific memory against *Mycobacterium bovis* bacillus Calmette—Guérin (BCG), the only currently licensed vaccine for preventing tuberculosis. In the present study, we investigated whether murine splenic NK cells can be activated by BCG in a dendritic cell (DC)-independent or -dependent manner, and furthermore examined whether these NK cells acquire specific memory following BCG vaccination. NK cells isolated from spleens of BCG-immunized mice produced interferon (IFN)γ through direct BCG stimulation in the absence of antigen-presenting cells; however, NK cells from control animals similarly directly responded to BCG, and the response level was not statistically significant between the immunized and the naïve NK cells. When purified NK cells that had been exposed to BCG were cocultured with RAW murine macrophages infected with BCG, the antibacterial activity of the macrophages was strongly enhanced; however, its level was similar to that by naïve NK cells, which had not been exposed to BCG. When splenocytes harvested from BCG-immunized mice were stimulated with purified protein derivative (PPD) derived from *Mycobacterium tuberculosis*, a specific IFNγ response was clearly observed, mainly attributed to NK cells and memory CD4^+^ T cells. To investigate whether these NK cells as well as the T cells are activated by cell−cell interaction with DCs presenting mycobacterial antigens, NK cells isolated from BCG-immunized mice were cocultured with splenocytes harvested from naïve mice in the presence of PPD stimulation. However, no IFNγ response was found in the NK cells. These results suggest that murine splenic NK cells do not develop BCG-specific immunological memory in either a DC-independent or -dependent manner.

## Introduction

*Mycobacterium tuberculosis*, the etiological agent of tuberculosis, primarily infects macrophages and dendritic cells (DCs). Replication of the bacteria is hampered by interferon (IFN)γ and tumor necrosis factor (TNF)-α, which are supplied by antigen-specific T cells and innate immune lymphocytes after the infection [1–4]. IFNγ in particular plays an important role in inducing resistance to *M*. *tuberculosis* infection. Indeed, it has been reported that mice in which the IFNγ gene has been deleted are much more susceptible to the infection than wild-type mice [1, 2]. As a mechanism of resistance by IFNγ to the infection, it is generally believed that after infection, activation of CD4^+^ T cells by mycobacterial antigens results in clonal expansion and the production of IFNγ, which activates macrophages resulting in their becoming mycobactericidal. In addition, the IFNγ has been shown to induce CD8^+^ T cell-mediated protective immunity against the bacteria in mice [5]. IFNγ is produced by natural killer (NK) cells as well as CD4^+^ and CD8^+^ T cells. While T cells exert the induction of acquired immune responses, NK cells are considered to contribute to evoking early protective immunity against many intracellular pathogens because of their ability to produce IFNγ during innate immune responses [6–10]. However, the role of NK cells in contributing resistance to intracellular bacterial infections including *M*. *tuberculosis* remains poorly understood [11, 12].

Recently, several lines of evidence have suggested that NK cells possess immunological functions similar to T cells [13–17]. It was first reported that NK cells can develop immunological memory as well as T cells in a hapten-induced contact hypersensitivity model using mice [18–20]. In addition, it has been shown that memory NK cells are elicited by viral infections such as influenza, vaccinia virus, vesicular stomatitis virus, genital HSV-2, human immunodeficiency virus type 1, and mouse cytomegalovirus [19, 21–24].

Recent studies showed that human NK cells are able to infiltrate granulomatous pulmonary lesions of tuberculosis [25] and that NK cells in pleural fluid from tuberculosis patients express the memory-associated marker CD45RO [26]. However, there is no direct evidence that NK cells induce mycobacterial antigen-specific, immunologically functional memory. In the present study, we investigated whether NK cells develop specific memory after vaccination with *Mycobacterium bovis* bacillus Calmette—Guérin (BCG), the only currently licensed vaccine for preventing *M*. *tuberculosis* infection, and furthermore examined whether BCG-sensitized NK cells provide enhanced immune responses in a DC-independent or -dependent manner. Because T cells residing in spleens of BCG-vaccinated mice are able to effectively develop specific memory, we focused on NK cells residing in the immunized spleens and compared the mycobacterial antigen-specific IFNγ response of the NK cells to that of the T cells.

## Materials and Methods

### Mice and cell lines

This study was approved by the ethics committee for biosafety and animal experiments of the Chiba Institute of Technology, Chiba, Japan. Female BALB/c and C57BL/6 mice of 4-weeks-old (Nippon SLC, Shizuoka, Japan) were maintained in a biosafety level two animal facility at the Chiba Institute of Technology. The animals were monitored every other day, and no unexpected deaths were observed. The animals were euthanized using isoflurane anesthesia (Intervet, Osaka, Japan) and the spleens were harvested. Macrophages of the RAW264.7 murine macrophage cell line (American Type Culture Collection ATCC; Manassas, VA, USA) were cultured at 37°C in RPMI-1640 (Sigma-Aldrich, St. Louis, MO, USA) supplemented with 10% fetal calf serum (Invitrogen), 100 U/mL penicillin, and 100 μg/mL streptomycin (Sigma-Aldrich).

### Immunization of mice with *Mycobacterium bovis* BCG

The BCG substrain Tokyo 172 (Japan BCG Laboratory, Tokyo, Japan) was grown at 37°C in Middlebrook 7H9 broth (BBL Microbiology Systems, Cockeyville, MD, USA) supplemented with albumin-dextrose-catalase (BBL Microbiology Systems) and stored in aliquots at −80°C until use. Four-week-old female C57BL/6 mice were immunized by a single intradermal administration of BCG (0.1 mg) or phosphate-buffered saline (PBS) as a control (*n* = 5 per group). The efficacy of the BCG vaccination was first confirmed by measuring mycobacteria-specific IFNγ responses in splenocytes of immunized mice.

### Preparation of NK cells

Spleens were harvested from naïve mice, or from mice vaccinated with BCG or PBS at 6 weeks after the immunization. Splenocytes were gently homogenized by passing them through a 70-μm nylon cell strainer (BD Falcon, Franklin Lakes, NJ), and the preparations were treated with red blood cell lysis buffer (Sigma-Aldrich) for 1 min at room temperature. NK cells were then isolated from the splenocytes by negative selection using the NK cell isolation kit II (Miltenyi Biotec, Bergisch Gladbach, Germany) according to the manufacturer’s instructions. The NK cells were cultured in RPMI-1640 supplemented with 10% fetal calf serum, 100 ng/mL murine interleukin (IL)-2 (Miltenyi Biotec), 100 U/mL penicillin, and 100 μg/mL streptomycin. The purity of NK cells assessed by fluorescence-activated cell sorting (FACS) was >85%.

### Analysis of cytokine production

To assess whether NK cells are directly activated by BCG or purified protein derivative (PPD) antigen, NK cells isolated from spleens of naïve mice were cultured (1.5 × 10^6^ cells/mL) in the presence or absence of BCG at multiplicity of infections (MOI) of 1 or PPD (50 μg/mL, Japan BCG Laboratory) at 37°C for 24 h, after which the culture supernatants were harvested. The PPD used consists of protein-enriched mycobacterial components manufactured from cultures of *M*. *tuberculosis* and is being widely employed as an antigen for a tuberculin skin test to diagnose tuberculosis infection. To further investigate whether NK cells can develop mycobacteria-specific memory, purified NK cells (1.5 × 10^6^ cells/mL) or total splenocytes (2 × 10^7^ cells/mL), both of which were obtained from BCG-immunized and PBS control mice, were stimulated with BCG (MOI = 1) or PPD (50 μg/mL) at 37°C for 24 h, after which the culture supernatants were harvested. Unstimulated cells were additionally prepared as a control. The production level of IFNγ was measured using the enzyme-linked immunosorbent assay (ELISA) kit according to the instructions of the manufacturer (eBioScience, San Diego, CA, USA). After harvesting a portion of the culture supernatants, brefeldin A (10 μg/mL; Sigma-Aldrich) was added to the remaining cell cultures for intracellular cytokine detection during the last 6 h of culture before harvesting the cells. The cells were blocked followed by being labeled with anti-mouse CD16/32 and PE-conjugated anti-mouse NK1.1, PE-conjugated anti-mouse CD4, or PE-conjugated anti-mouse CD8 monoclonal antibodies (mAbs), respectively (all eBioScience), permeabilized with cytofix/cytoperm solution (BD Biosciences, San Jose, CA, USA), and then stained with fluorescein isothiocyanate (FITC)-conjugated anti-mouse IFNγ mAb (eBioScience). The cells were analyzed on a FACSCalibur flow cytometer (BD Biosciences) and the data were analyzed using FowJo vX.0.7 (TreeStar, San Carlos, CA, USA).

To determine whether NK cells are activated through the mycobacterial antigen presentation by DCs, NK cells were isolated from the spleens of BCG-immunized and PBS control mice, and these cells were then cocultured with splenocytes harvested from naïve mice in the presence or absence of PPD stimulation (50 μg/mL) at 37°C for 24 h. The NK cells (3 × 10^5^) isolated from one mouse were mixed with the splenocytes (3 × 10^7^) obtained from one naïve mouse. The detection of IFNγ-producing NK cells was similarly performed by FACS as described above.

### Measurement of bacterial loads

RAW 264.7 murine macrophage cells were infected with BCG (MOI = 3) at 37°C for 2 h, washed three times with PBS, and then plated at 1 × 10^6^ cells/mL in a 12-well plate. Purified NK cells (3 × 10^5^), which had been stimulated with BCG (MOI = 1) at 37°C for 4 h, were added to the BCG-infected RAW cell cultures. As controls, unstimulated naïve NK cells were added to the BCG-infected RAW cell cultures, and the BCG-infected RAW cells were additionally prepared. Forty eight hours later, these cells and culture supernatants were harvested. The cells were lysed with 1 mL of a 0.067% sodium dodecyl sulfate (SDS) solution, and serial dilutions were plated on Middlebrook 7H10 agar plates containing oleic acid-albumin-dextrose-catalase (BBL Microbiology Systems). Three weeks later, the numbers of colony forming units (cfu) in the undiluted solution were determined. On the other hand, the culture supernatants were centrifuged at 9,000 × g for 5 min to remove BCG that might be slightly contaminated, and the production levels of IFNγ and TNF-α were measured using ELISA kits (eBioScience). To investigate whether NK cell activation required cell‒cell contact with BCG-infected RAW cells, purified naïve NK cells were cultured in medium supplemented either with the culture supernatant of the BCG-infected RAW cells or uninfected control RAW cells at a ratio of 1:1 at 37°C (1.5 × 10^6^ cells/mL). Twenty four hours later, the culture supernatants were harvested, and the production level of IFNγ was measured using ELISA.

### Statistical analyses

We conducted one-way analysis of variance (ANOVA) followed by the Tukey test for pairwise comparison, *t* test, or the Mann—Whitney *U* test; all calculations were performed using the Statistica program (StatSoft, Tulsa, OK, USA). The results are presented as mean ± standard deviation (SD); *p* values < 0.05 were considered statistically significant.

## Results

### Murine splenic NK cells are directly activated by BCG and PPD antigen

**Fig 1:**
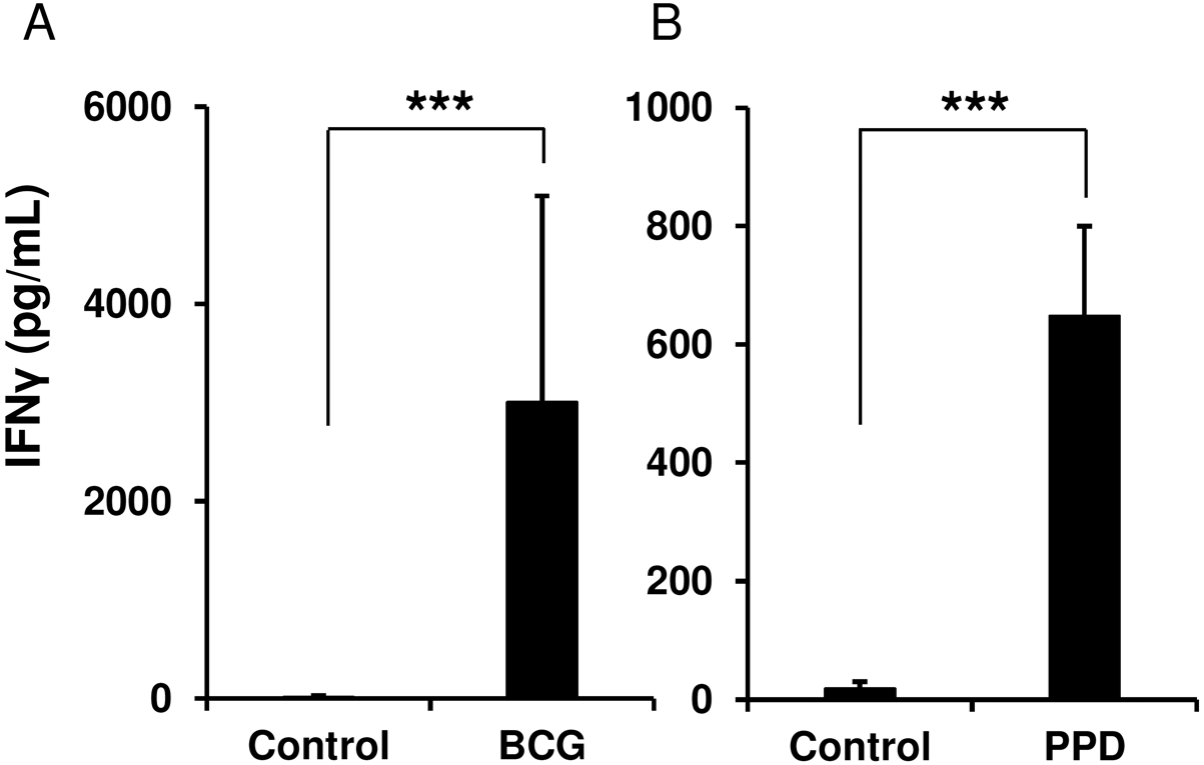
Spleen-resident natural killer (NK) cells are directly activated by *Mycobacterium bovis* BCG and purified protein derivative (PPD) antigen. NK cells isolated from spleens of naïve mice were cultured (1.5 × 10^6^ /mL) in the presence or absence of BCG (multiplicity of infections (MOI) = 1) (A) or PPD (50 μg/mL) (B) at 37°C for 24 h, and then the culture supernatants were harvested (*n* = 5 per group). The production level of IFNγ was measured by an enzyme-linked immunosorbent assay (ELISA). The data are presented as mean ± standard deviation, and *p* values < 0.05 were considered statistically significant. Similar results were obtained in three independent experiments. ***p < 0.0001.

To assess whether murine splenic NK cells can be directly activated by BCG and PPD antigen in an antigen-presenting cell (APC)-independent manner, we isolated NK cells from spleens of naïve mice, stimulating them either with BCG or PPD for 24 h, and then measured the level of IFNγ produced in the culture supernatants as an activation marker. Despite the absence of APCs, the purified NK cells were directly activated by BCG and PPD and produced IFNγ, whereas these cells were not activated by IL-2 alone (*p* < 0.0001, vs. unstimulated NK cells, Fig 1A and 1B). Concurrently, no IL-12 was detected in the culture supernatants, indicating that the NK cell activation observed should not be attributed to stimulation with cytokines by DCs, which might be slightly contaminated in the NK cell-rich preparations (data not shown).

**Fig 2:**
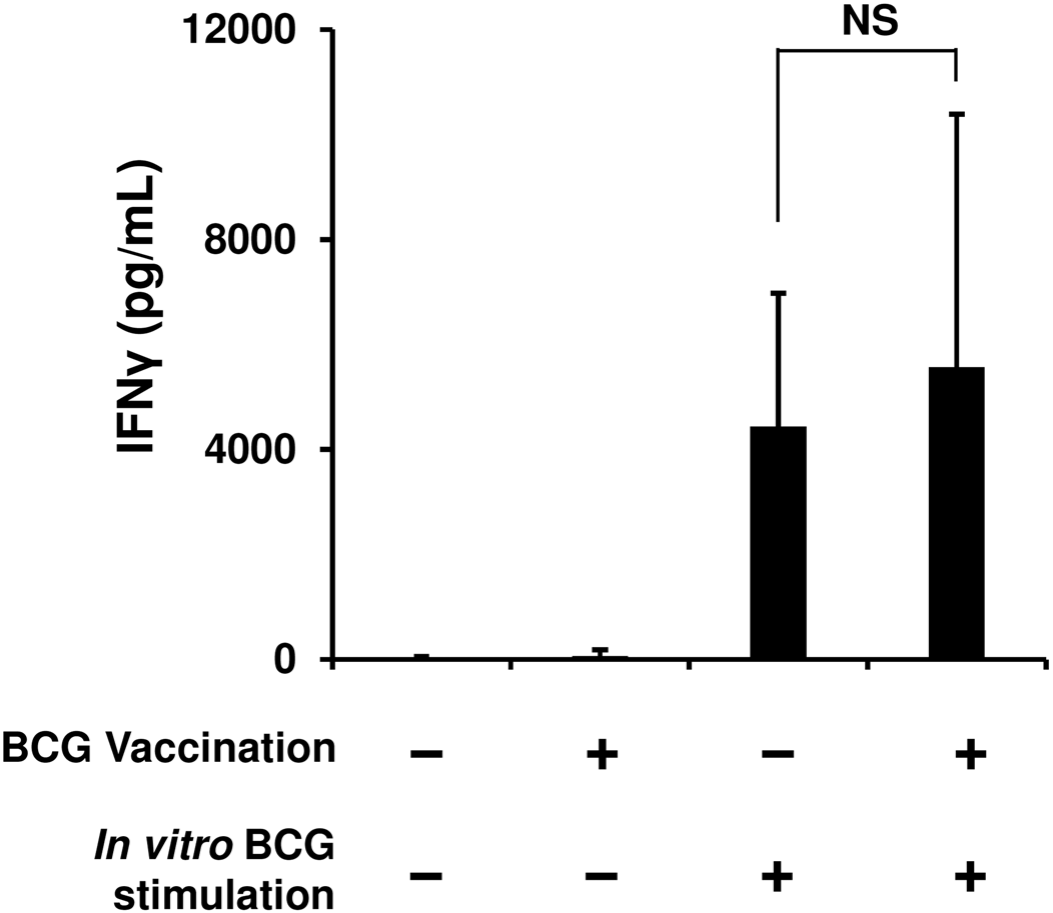
BCG vaccination does not enhance specific IFNγ production of NK cells in response to a second BCG stimulation. Mice were immunized with BCG or phosphate-buffered saline (PBS), and 6 weeks later, NK cells were isolated from the spleens of these animals (*n* = 5 per group). The purified NK cells were cultured (1.5 × 10^6^ /mL) in the presence or absence of BCG (MOI = 1) at 37°C for 24 h, and then the culture supernatants were harvested. The production level of IFNγ was measured by ELISA. The data are presented as mean ± standard deviation, and *p* values < 0.05 were considered statistically significant. Similar results were obtained in three independent experiments. NS, not significant.

To investigate whether murine splenic NK cells develop BCG-specific memory, we immunized mice with BCG or PBS as a control, and then isolated NK cells from spleens of these animals six weeks post-vaccination. When these cells were stimulated *in vitro* for 24 h using BCG, IFNγ was detected in the culture supernatants of both the immunized and control NK cells; however, the detected level of this cytokine in both the cells was statistically insignificant (Fig 2).

### NK cells markedly enhance the ability of APCs to eradicate BCG

**Fig 3:**
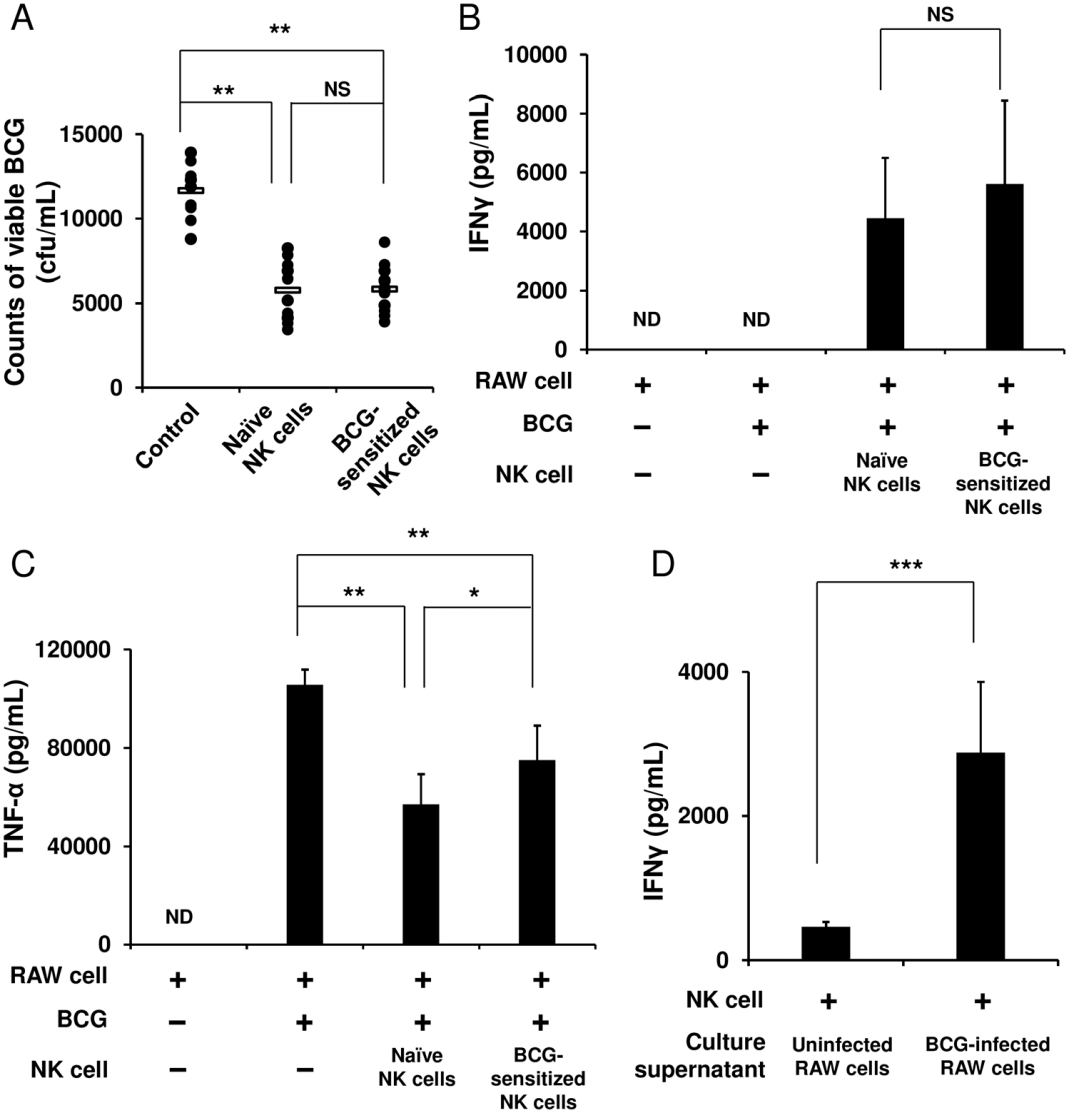
NK cells markedly enhance the ability of macrophages to eradicate BCG. RAW 264.7 murine macrophage cells were infected with BCG (MOI = 3) at 37°C for 2 h, washed with PBS three times, and then plated at 1 × 10^6^ cells/mL in a 12 well plate. Purified NK cells (3 × 10^5^), which had been stimulated with BCG (MOI = 1) at 37°C for 4 h, were added to the BCG-infected RAW cell culture. As controls, unstimulated naïve NK cells were added to the BCG-infected RAW cell culture, and the BCG-infected RAW cells alone were additionally prepared. Forty eight hours later, these cells were harvested and lysed with 1 mL of 0.067% SDS solution. Serial dilutions were plated on Middlebrook 7H10 agar plates, and 3 weeks later, the number of bacterial colonies grown on the agar plates were counted (A). As in (A), IFNγ (B) and TNF-α (C) in the culture supernatants were measured by ELISA. Purified naïve NK cells were cultured in medium supplemented with either the culture supernatant of the BCG-infected RAW cells or uninfected control RAW cells at a ratio of 1:1 at 37°C for 24 h, and IFNγ in the culture supernatants was measured using ELISA (D). The data are presented as mean ± standard deviation, and *p* values < 0.05 were considered statistically significant. Similar results were obtained in three independent experiments. Horizontal bar in (A), mean value; *p < 0.05; **p < 0.01; ***p < 0.0001; NS, not significant.

We next hypothesized that NK cells that once experienced BCG exposure may stimulate BCG-phagocytosed APCs more strongly than naïve NK cells that did not receive the exposure would. To test this hypothesis, the purified NK cells were first stimulated with BCG for 4 h and then cocultured with RAW 264.7 murine macrophage cells infected with BCG. After 48 h, these cells were harvested, and then from these cells, live BCG organisms were recovered. The amount of BCG in the RAW cells cocultured with the sensitized NK cells markedly decreased to about half the burden in the BCG-infected RAW cells alone (5,825 ± 1,488 vs. 11,660 ± 1,599 cfu/mL, *p* < 0.01, Fig 3A). However, this enhanced activation of the RAW cells by the BCG-sensitized NK cells was equivalent to that by the naïve NK cells (5,825 ± 1,488 vs. 5,773 ± 1,775 cfu/mL, Fig 3A). In addition and consistent with this result, the same level of IFNγ was detected in the two groups of NK cells (Fig 3B). On the other hand, with regard to TNF-α engendered by the BCG-infected RAW cells, the level of production significantly reduced when the RAW cells were cocultured with the naïve or BCG-sensitized NK cells from that in the RAW cells alone (Fig 3C). Furthermore, to investigate whether NK cells require cell−cell contact with BCG-infected APCs for NK cell activation, the culture supernatant of the BCG-infected RAW cells was added to the NK cell culture. As a result, the purified naïve NK cells were activated by the culture supernatant to produce IFNγ (*p* < 0.0001, Fig 3D), indicating that the IFNγ response of the NK cells should be elicited through the stimulation with cytokines secreted by the BCG-infected RAW cells, rather than by cell−cell contact between such RAW cells and NK cells.

### Vaccination of mice with BCG strongly induces IFNγ production in splenocytes

**Fig 4:**
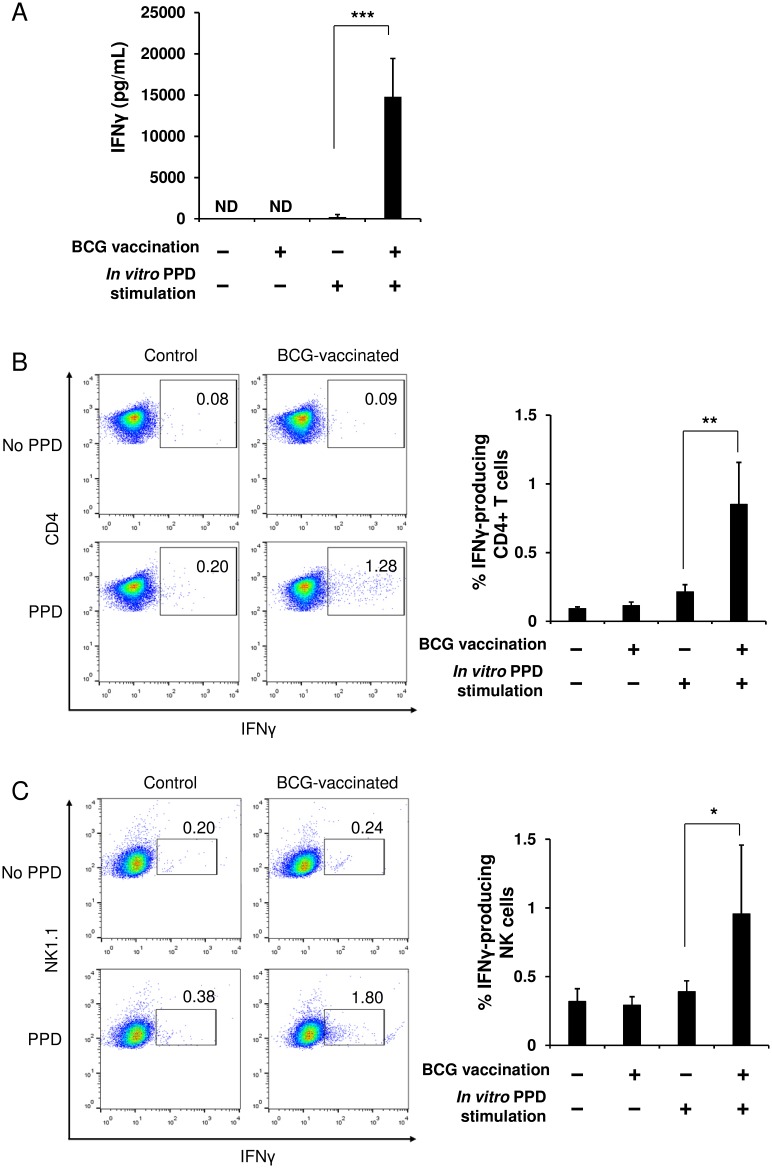
T cells and NK cells in spleens of BCG-immunized mice provoke specific IFNγ responses upon stimulation with PPD antigen. At 6 weeks after the single vaccination of mice with BCG or PBS, splenocytes were harvested from the immunized and the control mice (*n* = 5 per group). The cells were cultured (2 × 10^7^ cells/mL) in the presence or absence of PPD (50 μg/mL) at 37°C for 24 h. A portion of the culture supernatants was harvested for ELISA, and then brefeldin A (10 μg/mL) was added to the remaining cell cultures. The production level of IFNγ in the culture supernatants was measured by ELISA (A). The splenocytes harvested were stained with anti-mouse CD4 (B) or anti-mouse NK1.1 (C) followed by anti-mouse IFNγ mAbs, and then analyzed with flow cytometry. The data are presented as mean ± standard deviation, and *p* values <0.05 were considered statistically significant. Similar results were obtained in three independent experiments. *p < 0.05; **p < 0.01; ***p < 0.0001.

The experiments described in Figs 1–3 were performed using the purified splenic NK cells to examine the direct relationship between NK cells and BCG. Therefore, we next investigated an immunological relationship between NK cells, T cells, and DCs. When splenocytes were harvested from mice immunized with BCG or PBS and then stimulated *in vitro* with PPD, IFNγ was robustly produced by the splenocytes from the BCG-immunized mice; while it was not engendered by the splenocytes from the PBS control animals (Fig 4A). In addition, no IFNγ response was observed, even in the cells from the immunized animals if the *in vitro* PPD stimulation was absent (Fig 4A). These results clearly indicate that this IFNγ response observed in splenocytes of BCG-immunized mice is antigen-specific. Furthermore, flow cytometric analysis showed that CD4^+^ T cells and NK cells among the total splenocytes from the immunized mice produced IFNγ significantly in response to PPD stimulation, whereas they were unresponsive in the control animals (Fig 4B–4D). In contrast, little IFNγ response specific for PPD was found in the CD8^+^ T cells in either the control or the immunized mice (data not shown). These results suggest that IFNγ observed in the splenocytes from the immunized mice was produced mainly by NK cells and BCG-specific memory CD4^+^ T cells.

### NK cells are effectively activated by stimulation with cytokines produced by BCG-specific memory T cells

**Fig 5:**
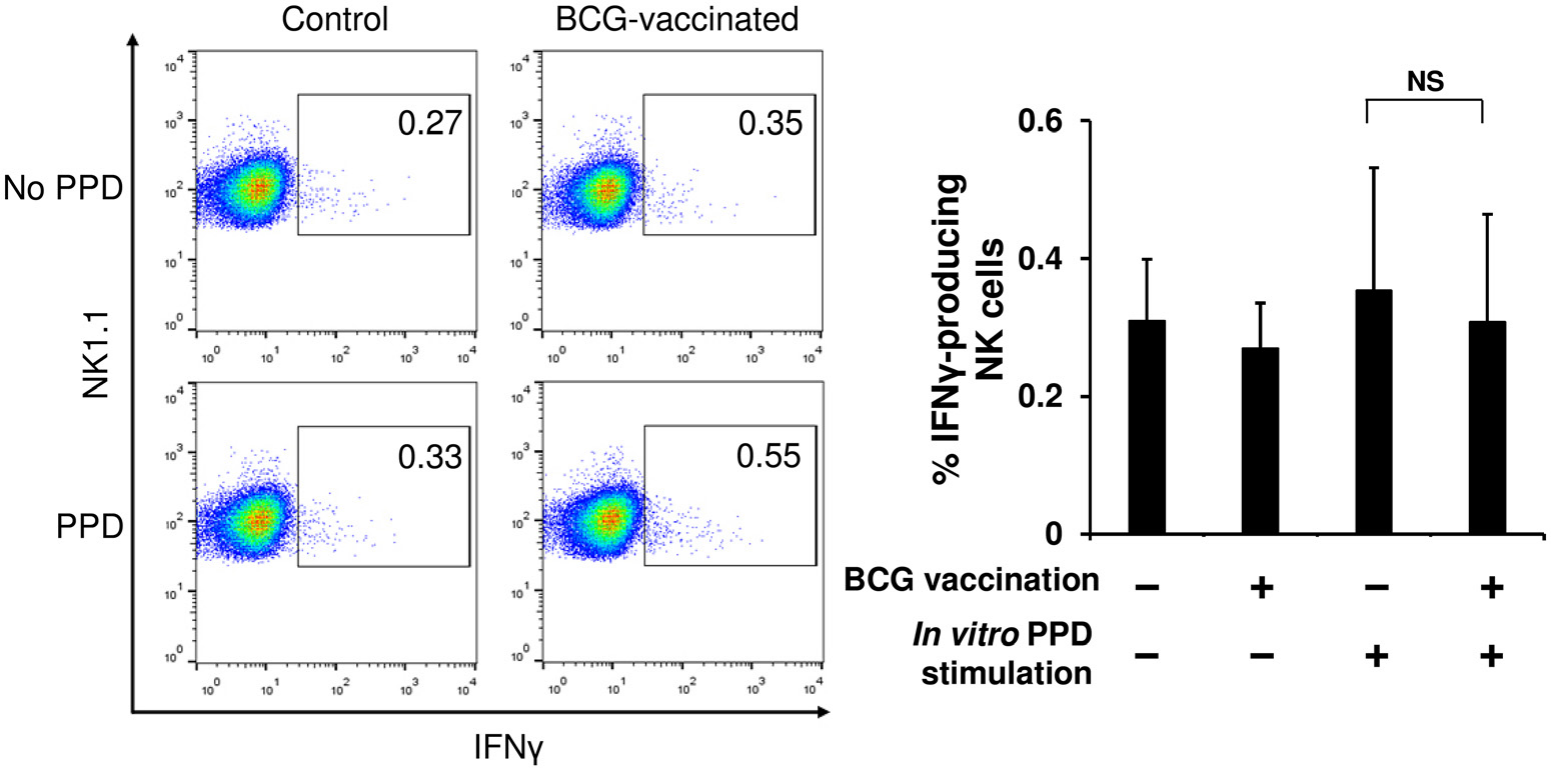
NK cells in spleens of BCG-immunized mice are not activated in a DC-dependent manner. At 6 weeks after the single vaccination of mice with BCG or PBS, NK cells (3 × 10^5^) were isolated from spleens of these animals, and then were cocultured with splenocytes (3 × 10^7^) harvested from naïve mice in the presence or absence of PPD stimulation (50 μg/mL) at 37°C for 24 h (*n* = 5 per group). The cells were stained with anti-mouse NK1.1 followed by anti-mouse IFNγ mAbs, and then analyzed with flow cytometry. The data are presented as mean ± standard deviation, and *p* values <0.05 were considered statistically significant. Similar results were obtained in two independent experiments. NS, not significant.

To investigate whether the IFNγ response of NK cells shown in Fig 4 was antigen-specific in a DC-dependent manner, NK cells were purified from the spleens of mice immunized with BCG or PBS, and then cocultured with splenocytes harvested from naïve mice in the presence or absence of PPD stimulation. In this experiment, the effect of cytokines produced by memory T cells in response to PPD on NK cell activation could be disregarded as no mycobacteria-reactive T cells exist in the spleens of naïve mice (Fig 4A), therefore an interaction between NK cells and DCs presenting PPD antigen is expected to be observed. As shown in Fig 5, IFNγ production in response to PPD was detected in neither the immunized NK cells cocultured with the naïve splenocytes nor the naïve NK cells cocultured with the naïve splenocytes, suggesting that unlike T cells, murine splenic NK cells cannot be directly stimulated through mycobacterial antigen presentation by DCs. This result indicates that the IFNγ response of NK cells observed in immunized splenocytes (Fig 4) may be evoked by cytokines produced mainly by BCG-specific memory CD4^+^ T cells rather than antigen presentation by DCs.

## Discussion

In the present study, we have demonstrated that: 1) purified murine splenic NK cells produced IFNγ through direct stimulation with BCG or PPD antigen in the absence of the mediation of APCs; 2) the magnitude of the BCG-induced IFNγ response of NK cells in the BCG-immunized mice was similar to that in PBS control animals; and 3) the activity of RAW macrophage cells to eradicate BCG was remarkably enhanced by NK cells; however, the contribution of the NK cell to the event did not differ between the naïve and BCG-sensitized NK cells. These results suggest that murine splenic NK cells may not develop specific memory to BCG. Furthermore, we have verified that: 4) when splenocytes harvested from BCG-immunized or control mice were stimulated *in vitro* with PPD, the NK and CD4^+^ T cells in the immunized mice induced an IFNγ response, whereas they did not exhibit the response in the control animals; and 5) this IFNγ response of NK cells in the immunized mice was induced mainly due to cytokine stimulation by BCG-specific memory T cells, rather than due to antigen-recognition by DC−NK interaction. These results suggest that murine splenic NK cells might not recognize mycobacterial antigen-presentation by DCs.

Recent studies suggest the feasibility of the contribution of NK cells to acquired immunity against viral infections [21–24]. However, it remains unclear how NK cells engage in the induction of immune responses after BCG vaccination followed by *M*. *tuberculosis* infection. Is the contribution of NK cells mycobacteria-specific or nonspecific? To approach these questions, we isolated NK cells from the spleens of BCG-immunized and control mice and then compared the levels of IFNγ production in response to *in vitro* BCG stimulation. Unexpectedly, no statistically significant difference in the level of the responses was observed between these two NK cells (Fig 2). This result implies that NK cells appear not to remember the first encounter with BCG, suggesting that NK cells may not develop BCG-specific immunological memory. BCG and its cell wall skeletons have been identified as agonists of toll-like receptors (TLRs) 2 and 4, which are believed to be major innate immune sensors for *M*. *tuberculosis* infection [27–30]. Therefore, the purified NK cells may have been directly stimulated with BCG and PPD via TLRs 2 and 4 (Figs 1 and 2) because NK cells have been shown to express these receptors [28, 31, 32].

It has been shown that neutrophils and DCs phagocytosed BCG are found at vaccination sites 4 h after intradermal BCG immunization in a mouse model [33]. During the first 4 h after intradermal BCG immunization, NK cells may directly encounter BCG until the uptake of the bacteria by APCs, which might sensitize these NK cells. If such sensitized NK cells should encounter the BCG-phagocytosed APCs that would correspond to a second encounter with BCG, the NK cells might provoke the strong activation of the APCs. To approach this, we assessed the enhanced ability of APCs to eradicate BCG by the assistance of NK cells. RAW 264.7 murine macrophage cells were strongly activated by NK cells that had received BCG exposure over 4 h, so that the RAW cells eradicated BCG more efficiently (Fig 3A). However, the magnitude of this enhanced ability to degrade BCG was equivalent to that conferred by naïve NK cells that had not received the previous BCG exposure (Fig 3A). In addition, IFNγ production between the BCG-sensitized and the naïve NK cells was almost at the same level (Fig 3B). These results suggest that splenic NK cells markedly enhance the ability of APCs to eradicate BCG; however, the efficacy of NK-cell assistance is not elevated, even if the NK cells experience direct BCG exposure prior to an encounter with BCG-infected APCs. Furthermore, when these purified NK cells stimulated with BCG for 4 h were washed and then further cultured for 48 h, no IFNγ was detected in the culture supernatants (data not shown). Considering that NK cells exhibited the IFNγ response after the stimulation with BCG for 24 h (Fig 1), induction of the response may require long-term BCG exposure. It appears to be impossible for NK cells to continue to receive such direct long-lasting BCG exposure *in vivo* because of the uptake of BCG by APCs [33]. Since NK cells were activated to a high degree by the culture supernatant of BCG-infected RAW cells (Fig 3D), NK cells should be more readily and rapidly activated via cytokines produced by the infected APCs, rather than via direct BCG exposure or cell−cell contact with the infected APCs. In particular, the NK cell activation observed in the current study is likely attributed to TNF-α produced by the BCG-activated RAW cells, as no IL-12 was detected in the culture supernatants. Intriguingly, by mixing the naïve NK cells or the BCG-sensitized NK cells with the BCG-infected RAW cells, the TNF-α level produced by the RAW cells declined to nearly half the level of the BCG-infected RAW cells alone (Fig 3C). This was correlated to a large degree with the BCG loads recovered from these RAW cells (Fig 3A). Because BCG-infected RAW cells were robustly activated by NK cells, such RAW cells might have terminated TNF-α secretion immediately after completely eradicating the BCG. TNF-α has been shown to increase the activity of macrophages to phagocytose and to eradicate mycobacteria [34, 35]. Furthermore, it has been reported that the phagosome maturation (phagosome acidification and fusion with lysosomes) of macrophages is effectively enhanced by IFNγ, leading to the increased effectiveness of macrophages to eradicate mycobacteria [36–38]. In the current study, the IFNγ response of NK cells may have contributed to the effective induction of phagosome maturation, enabling the RAW cells to eliminate BCG more strongly.

Having demonstrated that purified splenic NK cells from BCG-immunized mice do not develop DC-independent specific memory, we next investigated whether splenic NK cells will instead acquire DC-dependent specific memory. When total splenocytes prepared from BCG-immunized mice were stimulated *in vitro* with PPD, both CD4^+^ T cells and NK cells clearly exhibited an IFNγ response (Fig 4). This IFNγ response of the CD4^+^ T cells should be attributed to the memory CD4^+^ T cells conferred by BCG vaccination as no IFNγ response was observed in the cells from control animals despite of PPD stimulation (Fig 4A). On the other hand, with regard to the IFNγ response of the NK cells detected in the BCG-immunized mice, we have rejected the possibility that the NK cells were directly activated by PPD, since no IFNγ response of the NK cells was observed in the splenocytes of the control mice, even subsequent to the PPD stimulation, although purified splenic NK cells can be directly activated by PPD, as demonstrated in Fig 1B. Therefore, the two possible mechanisms underlying the NK cell activation observed in the immunized splenocytes would be considered to be as follows: 1) the NK cells might have been activated by cytokines secreted by the BCG-specific memory T cells that were activated through PPD stimulation; and 2) similar to T cell immunity, the NK cells might have additionally developed specific memory after BCG vaccination, and then such memory NK cells might have been specifically stimulated through PPD antigen-presentation by DCs. To address these queries, we isolated NK cells from spleens of BCG-immunized or control mice, and then cocultured the NK cells with splenocytes harvested from naïve mice in the presence of PPD stimulation. Under this experimental condition, we can eliminate an effect of cytokines on the activation of NK cells, such as IFNγ secreted by the BCG-specific memory T cells, as no BCG antigen-reactive T cells exist in the spleens of naïve mice (Fig 4A). Interestingly, when cocultured with the naïve splenocytes in the presence of PPD, the immunized NK cells did not produce IFNγ. This result suggests that subsequent to the vaccination of mice with BCG, the activation of NK cells in spleens may occur mainly due to the stimulation with cytokines produced by BCG-specific memory T cells, rather than due to the antigen-recognition by DC−NK cell interaction. Hence, the observed response of the NK cells should not be antigen-specific but be nonspecific. Collectively, upon stimulation with PPD, CD4^+^ memory T cells conferred by BCG vaccination are antigen-presented by DCs, activated exclusively by the presentation, and then produce cytokines such as IFNγ, leading to the subsequent NK cell activation.
